# Conceptualizing a Nursing Model for Integration of Patient Engagement Into Perinatal Digital Health Development and Quality Assurance: A Critical Interpretive Synthesis

**DOI:** 10.1111/nin.70041

**Published:** 2025-06-29

**Authors:** Jennifer Auxier

**Affiliations:** ^1^ School of Nursing The University of British Columbia Vancouver British Columbia Canada

**Keywords:** critical research approaches, grounded theory, maternal health, nursing models, technology

## Abstract

This study examines current assumptions of digital transformation research in the perinatal context and constructs a nursing model through a critical interpretive synthesis. Perinatal digital transformation research is discussed and found to be lacking grounding in nursing concepts; nursing theory was integrated by examining data through the lenses of Woman‐ and Family‐Centered Care (Person‐centered Perinatal Care) and by applying Donabedian's Frame of quality assurance into the conceptual matrix. Here, iterative data collection occurred, initially through a scoping review examining the nature and range of perinatal digital health systems. Purposive sampling of empirical studies was conducted to saturate the data pool with all four attributes of patient engagement (access, personalization, therapeutic alliance, and commitment). Participatory action theory supported an abductive stage of analysis and informed pragmatic construction of the model. The model encompasses: (1) person‐centered intervention mapping; (2) integration of process evaluation through stakeholder and user consultation; and (3) co‐creation during real‐life testing. The steps of the model are constructed to align with best practices in participatory action research, while holding nursing models as the foundational theoretical basis. This grounding in nursing theory will support a nursing lens for future action research related to the development of perinatal digital health systems.

## 1 Introduction

Person‐centered practices in perinatal care are being increasingly demanded by new and expectant parents; however, the definition and implementation of person‐centered practices in perinatal care settings remain challenging due to fragmented maternity and neonatal nursing practice structures and organizational and system health inequities. In middle‐ and high‐income countries, attempts are being made to enact person‐centered care (PCC) through the mandating of woman‐ and family‐centered care. However, the implementation of PCC practices is becoming increasingly complex as perinatal health systems are experiencing radical transformation in digital health system deployment.

As technologies make it possible for location and workflow to shift in high‐ and middle‐income countries, it is important to understand how the digitalization of perinatal health systems is influencing the enactment of patient engagement within PCC nursing models of practice. Patient engagement is comprised of four attributes: access, personalization, the capacity for parents to commit to self‐care and management (commitment), and the presence of a strong therapeutic alliance between practitioners and clients (Higgins et al. 2017). Engagement attributes are essential focal points for observing PCC practices within perinatal digital health systems and have the potential to provide the theoretical foundation for the development of quality assurance of these systems in the future. A nursing model of development and quality assurance is lacking, and therefore, systematic nursing‐led action research related to digital transformation is yet to be realized.

Implementation and efficacy evidence for patient engagement integration into perinatal (person‐centered) digital health practices are few (Auxier, Bender, et al. 2023). Instances of impersonal, inaccessible, and unsatisfactory perinatal person‐centered care have been experienced by new and expectant parents with and without digital health system integration (Oelhafen 2024; Nyman et al. 2011; Platonos et al. 2018; Van Veenendaal et al. 2021; Wu et al. 2019). Literature in high‐ and middle‐income countries in the field of perinatal nursing reveals that new and expectant parents desire improved access to information from healthcare professionals (Lupton 2016), have experienced distress due to visiting restrictions during the events of the global SARS‐CoV‐2 pandemic, and have experienced interactions with nurses and midwives that are impersonal and isolating during labor (Koster et al. 2020; Ängeby and Ternström 2024). Person‐centered perinatal digital health is emerging in dynamic and nuanced ways, which is compounding the urgency of integrating person‐centered and engaging nursing care practices into digital health systems (Auxier, Bender, et al. 2023). Perinatal digital health practices were observed in a scoping review as multifaceted and highly complex interventions that lack transparency (Auxier, Bender, et al. 2023). The current rate of perinatal digital health development and deployment leaves little capacity for evidence‐based design and quality assurance, and the nursing perspective has been lacking (Auxier, Bender, et al. 2023).

Historically, new technologies in perinatal nursing have made it necessary for care users and providers to adapt. In these cases, it has often been challenging to assure quality in practice during dynamic transformations with the use of new technologies (Grönvall and Verdezoto 2013; Mol 2009). Perinatal digital health nursing is one form of “e‐scaped” care. “E‐scaped” care frames digital health practices as structures and processes of care influenced by sociomaterial factors. Not since the early 1800s (with the advent of increased medical technologies and expertise moving care from home to hospital) has such a shift in structure and location of healthcare been experienced around the world. The sense of responsibility parents feel for their own care and use of medical devices in home environments has increased (Anwar Siani et al. 2017; Auxier et al. 2022; Oelhafen 2024). Before ubiquitous digital health use, parents could only access reliable blood pressure readings and weights from clinic or hospital settings. Now it is possible to impact parental feelings of control and empowerment by giving them access to blood pressure self‐monitoring technologies that can function to automatically send health data to a server through Bluetooth technology directly to their nurses in real time (Hirshberg et al. 2018; Rhoads et al. 2017). Further, new parents can perform health assessments on their infants in the comfort of their own homes and send pictures to the nurses for follow‐up on transition to life at home after a neonatal intensive care (NICU) unit admission (Danbjørg et al. 2015). Now that perinatal care has “e‐scaped” how is quality of care defined, how can we best develop perinatal digital health nursing practices to meet the needs and demands of parents? As novel digital health strategies (i.e., self‐monitoring, remote communication, serious gaming, and generative artificial intelligence etc.) are already being employed, concerns about the possibility of losing an awareness of who is responsible for doing the caring work is relevant (Oudshoorn 2011). While perinatal digital health nursing is being tested for feasibility, usability and effectiveness in relation to clinical outcomes, the investigation into what processes of patient engagement impact effective and sustainable development and use of person‐centered and engaging digital health systems has not been extensively studied from a nursing perspective (Auxier, Bender, et al. 2023; Barello et al. 2016).

A systematic and evidence‐based tool for knowledge integration and dissemination of new and expectant parents' experiences, needs, and preferences for digital health interventions is lacking. A patient engagement model that integrates patient involvement and inclusion in design (action research), nursing models of care (woman‐ and family‐centered care), nursing theory (Donabedian's Quality assurance frame), and present modalities of perinatal digital health would inform holistic development and quality assurance of perinatal digital health.

## 2 Background

Because the location of care is transforming with ubiquitous use of smartphones, wearable devices, and generative artificial intelligence, the demands on patients to engage in the collection and interpretation of their health data is greater than ever before. Patient engagement has been touted as the blockbuster drug of the century by some (Dentzer 2013), however in a scoping review examining the integration of patient engagement within perinatal digital health practice and quality assurance it was shown that important elements of patient engagement, and nursing concepts are not being integrated into current digital health practices in perinatal nursing (Auxier, Bender, et al. 2023). The scoping review concluded that more research is needed to understand and examine emergent processes of parents' commitment/motivation in engagement in perinatal care and to explore what shapes the therapeutic relationships between nurses and parents in the context of digital health system use (Auxier, Bender, et al. 2023). The radical digital transformation in these care contexts can be conceptualized as the emergence of “e‐scaped” care (Nettleton 2004). If “e‐scaped” care is to support higher and beneficial levels of patient engagement in perinatal practice, a systematic way of integrating patient engagement as processes and structures during the development and evaluation of perinatal digital health systems is required.

**Figure 1:**
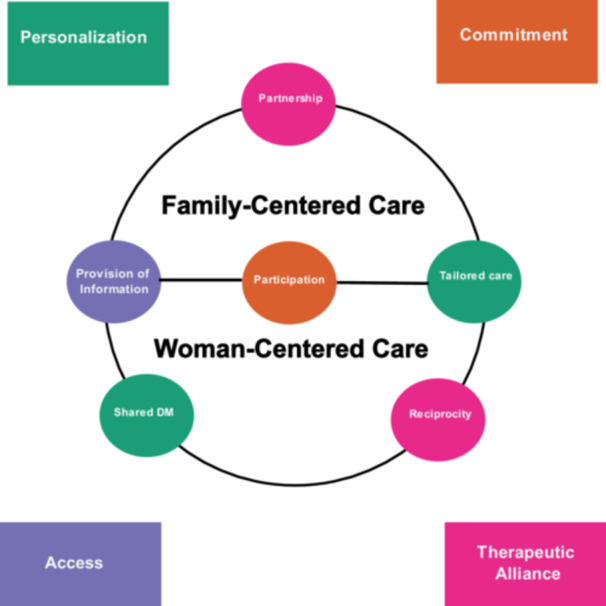
Mapping woman‐ and family‐centered care elements to the attributes of patient engagement: concepts adapted from patient engagement (Higgins et al. 2017); woman‐centered care (Fontein‐Kuipers et al. 2018); family‐centered care (Franck and O'Brien 2019). The colors of concepts are mapped to the patient engagement attributes.

Examining perinatal digital health systems that integrate patient engagement is useful in deconstructing the interactions and dynamic processes occurring within “e‐scaped” perinatal nursing care. The next step is to understand the link between patient engagement and essential concepts of family‐ and woman‐centered care. The definition of patient engagement outlines four attributes, and represents both processes and behaviors: access, personalization, commitment, and therapeutic alliance (Higgins et al. 2017). Woman‐ and family‐centered care contains core components of respect for personhood, patient involvement, collaboration, self‐care, and self‐management during the perinatal periods (Fontein‐Kuipers et al. 2018; Franck and O'Brien 2019). Some elements of woman‐ and family‐centered care can be mapped to the attributes of patient engagement (see Figure 1).

**Figure 2:**
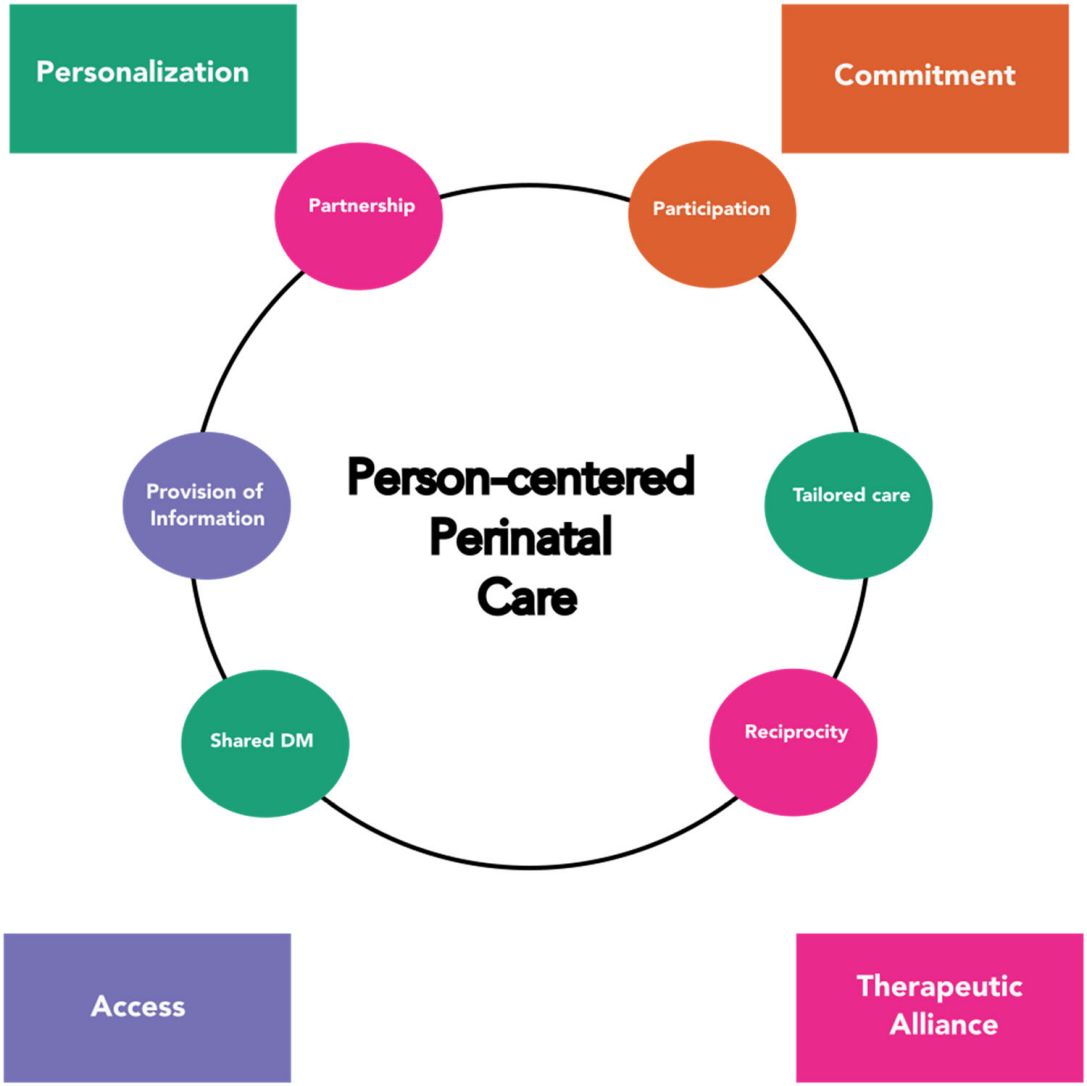
Woman‐ and family‐centered care are coupled with patient engagement attributes in the context of digital health practices to form parental perinatal digital practices.

A conceptual structure is needed to clearly articulate best practices for development and quality assurance in perinatal digital health nursing. It is possible to arrange our understandings of person‐centered perinatal digital health as a structure that has woman‐ and family‐centered care concepts at its core and relies on patient engagement principles for enacting person‐centered perinatal care (see Figure 2).

Registered nurses, the largest workforce within acute perinatal care, midwives, and obstetricians have been struggling in recent years to develop sustained practice of woman‐ and family‐centered care (Fontein‐Kuipers, Boele, et al. 2016; Stelwagen et al. 2020). Much attention has been placed on measuring effectiveness and efficacy of perinatal services, or medical and individual health behavior outcomes; such as adherence to breastfeeding, optimal management of hypertension, and gestational weight gain during pregnancy (Baruth et al. 2019; Hirshberg et al. 2018; Jefferson et al. 2019). While the evaluation of these outcomes is essential for maintaining and evaluating safety and health outcomes in perinatal programs, the evaluation of patient engagement practices and processes (i.e., woman‐ and family‐centered care) is a vital and core component of assessing quality.

When perinatal care programs are designed appreciating parents as unique and composite members of the care teams, parental self‐confidence and feelings of reassurance improve, and adverse outcomes for both parents and infants can be ameliorated (Ahlqvist‐Björkroth et al. 2017; Franck and O'Brien 2019; van Veenendaal and Limpens 2020). Recent research has illustrated that perinatal digital health use is positively impacting parents' perceptions of care delivery. Some parents have felt more connected to care services daily and liked having the chance to initiate the processes of their care by sending photos, requesting video calls, or viewing videos to train themselves in breastfeeding techniques on demand (Danbjørg et al. 2015; Jefferson et al. 2019; Shorey et al. 2018). While parent participation in healthcare has been associated with greater patient safety the optimal care, the impact of transferring care activities to parents with the use of digital health solutions should be examined (Danbjørg et al. 2015; Gibson et al. 2021; Platonos et al. 2018). The integration of patient engagement in digital health systems has the potential to stimulate parents emotionally and cognitively in their commitment toward maintenance of family wellness and self‐care activities in collaboration with their nurses/primary care providers (Auxier, Savolainen, et al. 2023; Rhoads et al. 2017). The quality of the provider‐patient interactions and the maintenance of mutual trust between providers and patients remain a vital component when integrating engagement into digital health systems (Gibson et al. 2021; O'Brien et al. 2013).

The structure of perinatal nursing care is transforming and integrating new care processes and interactions between nurses, parents, and digital health modalities (Auxier, Bender, et al. 2023). Care processes occurring within perinatal digital health influence a dynamic flow between stability and change due to the various new interactions occurring during the adoption and adaptation of new technologies (Oudshoorn 2011). The new insights gained through self‐monitoring by parents can be channeled into better optimization of sleep, physical activity, and stress reduction for health promotion of pregnant persons and their unborn babies (Auxier, Savolainen, et al. 2023; Auxier et al. 2022; Lupton 2017; Niela‐Vilén et al. 2021; Saarikko et al. 2020). Parents can also prepare for clinic visits or remote video calls differently with the help of on‐demand, evidence‐based perinatal information and access to personal data (Grönvall and Verdezoto 2013; Strand et al. 2022; Auxier et al. 2022). Parents are engaging in perinatal digital health systems; however, the level and scope of nursing research about the impact and success of the integration of measurable attributes of patient engagement in the design and quality assurance of these systems is lacking.

## 3 Aim

**Figure 3:**
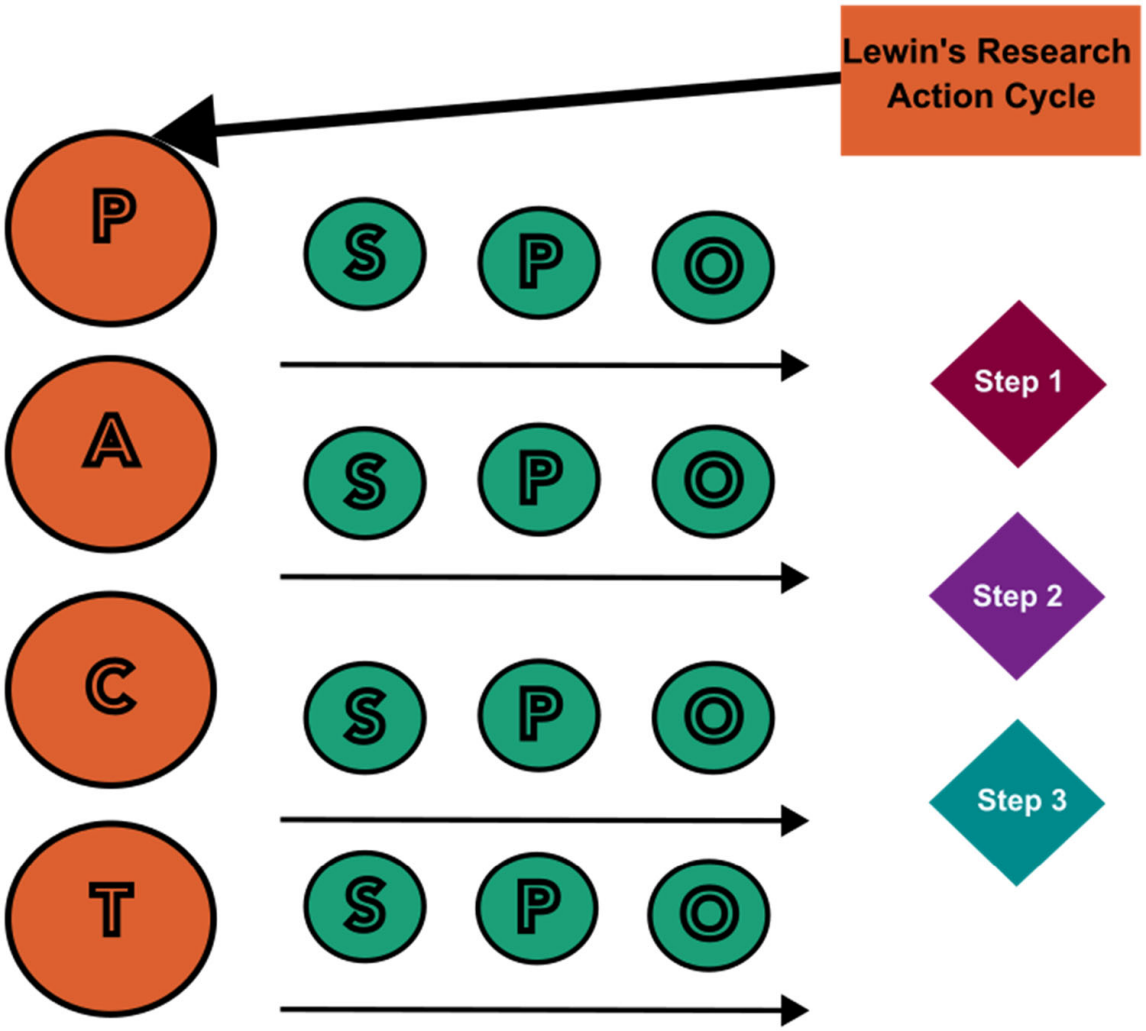
Conceptual matrix for grounded theory of study. PACT = the four attributes of patient engagement (Higgins et al. 2017); SPO = structure, process, outcome (Donabedian 2002).

A critical interpretive synthesis was conducted to aggregate and synthesize methodologically diverse perinatal digital health practice narratives and empirical data into a nursing model for patient engagement integration in perinatal digital health development and quality assurance. A model was generated using pre‐existing textual evidence related to perinatal patient engagement digital health practices (see Figure 3 and Table 2). The research question that guided this empirical study was: What are the steps of patient engagement integration in perinatal digital health development and quality assurance that make up a nursing engagement model?

## 4 Methods

This study was the fourth phase of a larger exploratory study that comprised empirical evidence examining the nature, range, and implications of the current states of patient engagement integration in design and quality assurance of perinatal digital health nursing in high‐ and middle‐income countries (Auxier, Bender, et al. 2023). Purposeful sampling of empirical sources was conducted, and these primary sources were combined through deductive and abductive reasoning, supported by the definition of patient engagement, Donabedian's framework for quality assurance, and an adapted version of Lewin's Action Research Cycle (Oberschmidt et al. 2022; Williamson et al. 2011). A critical interpretive synthesis (CIS) approach was employed through articulation of steps for patient engagement integration in perinatal digital health development and quality assurance in the form of a nursing model (Strauss and Corbin 2007; Thorne 2016). Data collection and selection methods for the initial 80 sources of this interpretive synthesis are reported in a scoping review elsewhere and make up the initial phase of this CIS (Auxier, Bender, et al. 2023).

### 4.1 Theoretical Foundations for a Model of Patient Engagement Integration

Three theoretical frames were applied in the construction of the patient engagement model. The first, was a deductive descriptive step using the four attributes of patient engagement (Higgins et al. 2017). The second, Donabedian's conceptual model for the assessment of quality care was incorporated in the second deductive phase of the analysis (see Table 1). Donabedian introduced the three approaches to quality assessment in their conceptual model first in 1966. The three approaches represent three dimensions of care to be considered when evaluating quality of care provision and are structure, process, and outcome (Donabedian 2002). The third theoretical frame is adapted from Lewin's 1946 Action Research Cycle and was incorporated into the synthesis of the patient engagement model in the abductive phase of the analysis. Lewin is credited for being the first to describe the Action Research Cycle and this conceptual model has been later explained by Williamson as an ideal method for changing workplace practice by putting emphasis on reflective processes that support the generation of new knowledge and understandings about the practice and quality of care (Williamson et al. 2011). The Action Research Cycle was further adapted by Oberschmidt and colleagues to reflect relevant integration of the cycle into digital health design and implementation practices (Oberschmidt et al. 2022).

**Table 1:** Analytical process for interpretive synthesis.

Theoretical frame	Methods and concepts of matrix
Patient engagement attributes(access, personalization, commitment, and therapeutic alliance) (Higgins et al. 2017)	Initial deductive and descriptive step of analysis (details published earlier; Auxier, Bender, et al. 2023).
Donabedian's Quality Assurance Frame(Donabedian 2002)	Perinatal digital health system structures, processes, and outcomes (SPO) were extracted and charted using the deductive approach according to each patient engagement attribute
Adapted version of Lewin's Action Research Cycle (Williamson et al. 2011)	Final deductive steps were taken by applying the previously extracted data─according to SPO categorization—into a matrix composed of four steps of the Action Research Cycle:
	Diagnose and plan
	↓
	Implement the action strategy
	**↓**
	Evaluate the action strategy
	**↓**
	Reflect plan again and “re‐spiral”

The construction of this nursing model was aimed to describe and provide a harmonized set of steps for nursing and research practice that allows the discrete workings of perinatal patients, and digital health modalities to be visualized through recording and evaluating processes and outcomes that will continually build and re‐design a structure of nursing and parental digital health practice. The kind of service provision aimed for is one that is personalized and flexible to change while maintaining core components of the structure (person‐centered perinatal care). The nature of a new perinatal system that integrates both digital health modalities and patient engagement principles could be one that moves across a spectrum of care models (woman‐ and family‐centered care) dependent on the preferences, actions, participation level, interactions, and collaboration of all actors (care context, parents, nurses, and digital health modalities) within the system. The three theoretical frames used in this study support the synthesis of a model that would allow for the examination and practice of systems in continual movement while maintaining the core components of person‐centered perinatal nursing care.

### 4.2 Data Collection and Selection Process

Data collection was iterative and was based on the findings and interpretations gained from the first three phases of the larger exploratory study. Inclusion and exclusion criteria of primary sources are reported in the original scoping review (Auxier, Bender, et al. 2023). A progressive interpretation of Donabedian's quality assurance model, and an adapted version of Lewin's Action Research Cycle developed specifically for digital health research (Oberschmidt et al. 2022) guided the CIS analysis. Further, existing conceptual frameworks of woman‐ and family‐centered care supported the conceptual fitting of a model for the practice realities of a perinatal nursing care context (Tavory and Timmermans 2014; Thorne 2016). The first phase of data collection included obtaining original research in the form of peer reviewed articles, and conference reports sampled through a systematic search published elsewhere (Auxier, Bender, et al. 2023). The findings of a scoping review conducted about the nature and range of patient engagement integration in perinatal digital health illustrated that little had been reported in the field about commitment, therapeutic alliance, and process evaluation of perinatal digital health nursing practices (Auxier, Bender, et al. 2023). Commitment and therapeutic alliance, process evaluation, and nursing conceptual underpinnings were then identified as the four important areas of discovery for the construction of a nursing action research practice model. Sources in phase two were selected because they reported on commitment, therapeutic alliance, and process evaluation related to person‐centered perinatal practices. See Table 2 for the description of source sampling.

**Table 2:** Sampling description of the interpretive synthesis.

Sampling rationale	Number of sources
Scoping review (Auxier et al. 2023).	*N* = 80
Purposive search for a collection of empirical studies about Therapeutic Alliance: Targeted Google Scholar search and consultation with experts	*N* = 2 (Niela‐Vilén et al. 2016; Wu et al. 2019, 2020)
Purposive search for collection of empirical studies about process evaluation: Pilot study of self‐monitoring perinatal digital health system and development of NICU parent participation process measure	*N* = 3 (Auxier et al. 2022; Auxier, Savolainen, et al. 2023; van Veenendaal et al. 2021)
**Total sources**	***N*** = **86**
**Sources relevant for the construction of model**	
Step 1	*N* = 85^a^
Step 2	*N* = 86
Step 3	*N* = 85^a^

^a^ Van Veenendaal et al. (2021) was not relevant for the construction of Steps 1 and 3.

### 4.3 Data Extraction, Charting, Analysis, and Synthesis

Literature in the field was purposively chosen at the beginning of the study to ensure the inclusion of information sources that related to the current state of person‐centered perinatal digital health development and quality assurance. All empirical data included in the larger exploratory study (Auxier 2023) were sampled. Data extraction was conducted using a deductive grounded theory approach guided by a conceptual matrix (see Figure 3) that had been developed through conceptual insights gained from the larger exploratory study. Data was charted according to study design, author, year, design development procedures, and the identified structure, process, and outcomes of each perinatal digital health system. The data were synthesized through an abductive phase where linkages were mapped and placed in three newly constructed recommendations referred to from here on as the steps that depict the nursing engagement model (Tavory and Timmermans 2014; Thorne 2016).

### 4.4 Rigor and Reflexivity

Trustworthiness and reflexivity were attended to in this study through the keeping of audit trails which contained consistent labeling of meaning units, stages of the conceptual matrix development, and consideration of the distant phases of deductive and abductive analysis (Thorne 2016). Further, research experts were consulted to test interpretations and conclusions based on their expertise in grounded theory and perinatal digital health development (Tavory and Timmermans 2014). The author has a background in perinatal nursing and worked closely with neonatal and newborn care experts (nurses and midwives) during the analysis and synthesis phases of this study to understand the differences and similarities of maternity and neonatal care objectives, for their clear integration into a perinatal engagement model.

### 4.5 Ethical Considerations

This study did not require accessing private details of individuals at any point in the study, and obtaining ethical approval was not required to conduct this study.

## 5 Results

**Figure 4:**
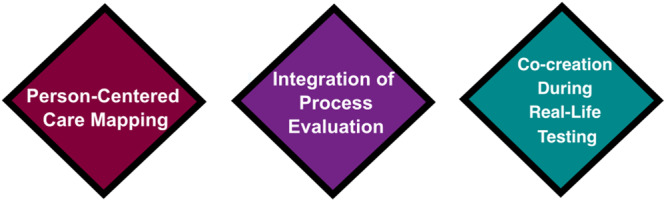
Three steps of the model for patient engagement integration in perinatal digital health development and quality assurance.

Interpretation and a data chart of all included empirical sources (see Table 2) were produced with the use of the Donabedian Quality Assurance model, and the attributes of patient engagement. Critical Interpretive Synthesis was employed through the use of a conceptual matrix (see Figure 3) and an identification of practice/theory gaps from the initial deductive descriptive step, abduction was harnessed to highlight the similarities between the included empirical sources (*N* = 86) and the Lewin's Action Research Cycle, and the fitting of person‐centered care and patient engagement practices. The structure of perinatal digital health systems was depicted in Step 1 of the model through the person‐centered mapping of digital health modalities to specific patient engagement attributes (access, personalization, commitment, and therapeutic alliance). Further, human‐centered design practices and process evaluation principles embedded in findings were charted to all four attributes of patient engagement and were constructed into pragmatic steps in the model. The model for patient engagement integration into perinatal digital health includes three steps: (1) person‐centered intervention mapping; (2) integration of process evaluation through stakeholder and user consultation; and (3) co‐creation during real‐life testing (see Figure 4). The following sections of this report describe the three steps in detail.

### 5.1 Step 1: Person‐Centered Care Mapping

**Figure 5:**
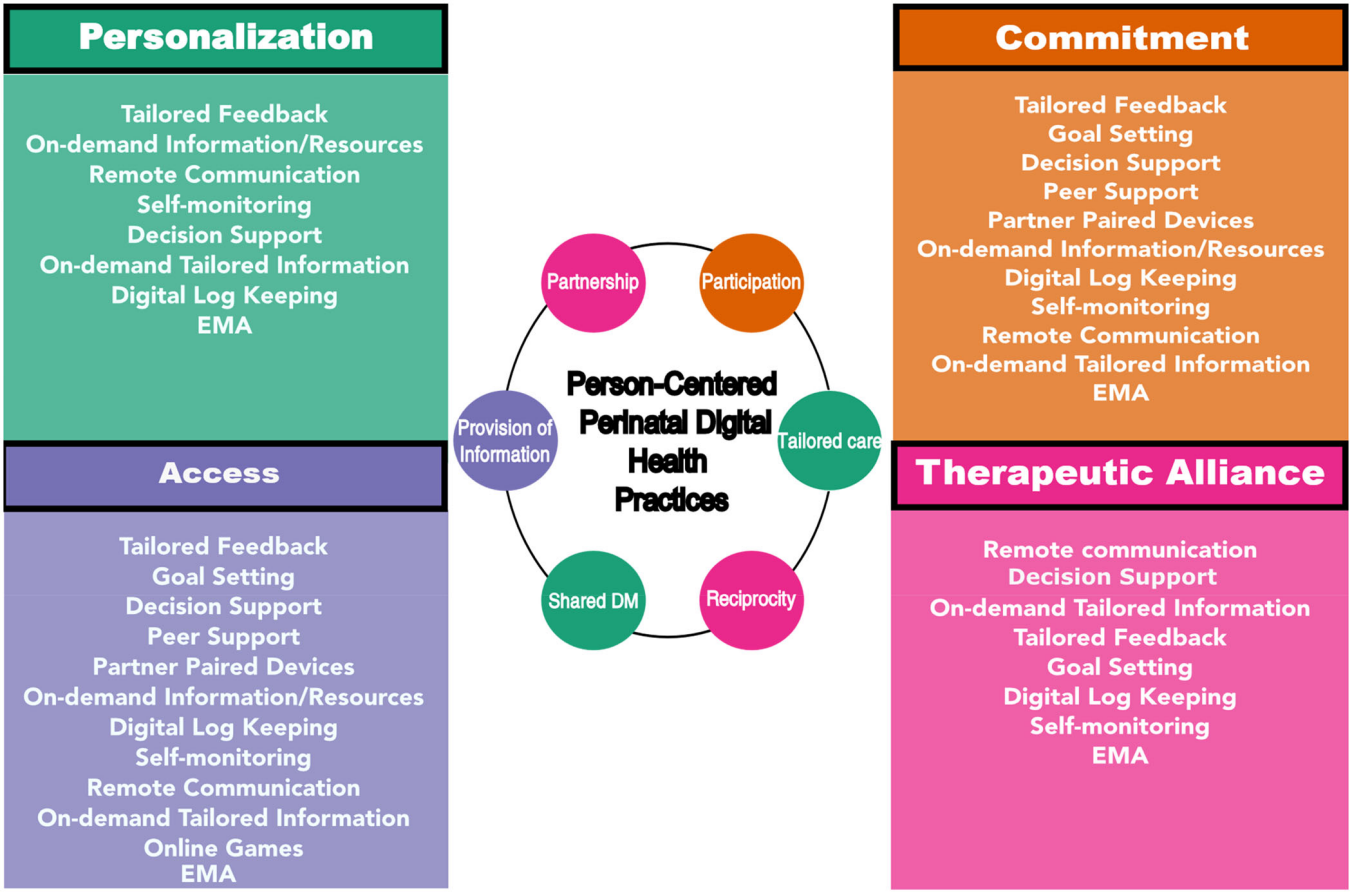
Taxonomy of perinatal digital health modalities mapped to attributes of patient engagement.

The first step of the model provides a starting point for the development and planning of meaningful perinatal digital health systems that will support person‐centered perinatal care structures and processes. The WHO recommends using a shared language for the development and classification of digital and mobile technologies used to support health system challenges. While the classification of digital health interventions (DHI) developed by the WHO exists, a taxonomy and template for mapping person‐centered health system challenges and DHIs is not explicitly highlighted. Digital health modalities and their descriptions were constructed from the scoping review analysis (Auxier, Bender, et al. 2023). The 12 digital health modalities in use in perinatal digital health interventions can be linked to health system challenges. Perinatal health system challenges are linked to limited or non‐sustained practice of the perinatal care core values of partnership, participation, provision of information, shared decision‐making, reciprocity, and tailored care (see Figure 5). The process of mapping perinatal health system challenges and digital health modalities is lacking in the current design and development research in this field. Here, a taxonomy and mapping template of person‐centered perinatal digital health modalities was interpreted through the concept of structure (Donabedian 2002). This taxonomy as a step in the model provides design logic for the integration of patient engagement attributes into the structure of perinatal digital health nursing interventions. This taxonomy could support identification of perinatal person‐centered health system challenges (World Health Organization 2018) and inform explicit design decisions.

A clear description of the perinatal health system challenges should be made at the outset of planning with nursing researchers and parents to guide developers and stakeholder advisory panels in their discussions about possible design features/digital health modalities (see Figure 5). The first step in this model can guide the clear conception of patient engagement elements to be integrated according to the deficiencies (health system challenges) that have been identified by perinatal nurse experts and parents.

### 5.2 Step 2: Integration of Process Evaluation Using Vignette Descriptions

The pilot study of a self‐monitoring perinatal digital health solution and the development of a process measure in the NICU provide the theoretical foundation for placing process evaluation determination as the focus of Step 2 of this model (see Table 2). Important patient engagement processes were observed during technology use, adaptation, and parent partnership practices. These processes are complex and require examination of the nuanced practices of parents and perinatal nurses. Here, Step 2 is depicted as a vignette description and illustrates a possible process evaluation approach and associated workflow that could be developed from the initial findings from Step 1 (person‐centered intervention mapping).

This vignette is intended to be used in future participatory action research to elicit stakeholder views and preferences of a theoretical digital health system. In what follows is a description of a hypothetical digital health system for use by nurses and parents of newborns experiencing care in the NICU after traumatic birth or illness in a fictional city, so named, City P. This vignette description includes information about the structures and processes that were theoretically mapped using Step 1 of the engagement model. The vignette follows:

The hospital in City P has integrated woman‐ and family‐centered care values in their operations and policies in the perinatal and NICU units and has been certified as a baby‐friendly hospital. City P hospital received negative feedback from the yearly health user survey related to person‐centered care. The feedback was:

- Parents feeling that care was often not personalized.
- Parents noticed that they were missing important information and updates about their newborns' health status when parents were away from the unit.
- Parents feel unprepared to return home with their newborns.

A committee of parents, parent support organization liaisons (Ronald McDonald House), nurses, and neonatologists was formed to tackle the quality improvement for increased practices of person‐centered perinatal care. The committee initiated the engagement model and after multiple consultation sessions and person‐centered mapping rounds (Step 1 of the engagement model) the CO‐PARTNER parent health engagement system was designed as a vignette case.

The structure of the CO‐PARTNER parent health engagement system includes five digital health modalities aimed at supporting increased personalization and access to perinatal care resources/information. The five modalities include the following:

- Remote communication
- On‐demand digital information and Resources
- On‐demand tailored feedback
- Digital log keeping
- Tailored feedback (synchronous)

The CO‐PARTNER parent health engagement system includes an App that parents can download on either iOS or Android smartphones. The App is only available through the health authority and the region of City P hospital. The App is available as a mobile version and a web version (allowing for nurses and other health professionals to access data and information using a desktop or laptop computer). The App includes a professional communication section which allows parents to chat and make appointments with healthcare providers (in‐person or remote appointments). The parents can chat with nurses from the NICU using asynchronous messaging (SMS or email). There is an evidence‐based library and searchable database that includes videos and health information that have been sourced through trusted organizations and universities in the region. Parents complete a personal family profile when they are admitted to the NICU or perinatal units. Parents can include the date of delivery, gestational age of the newborn at the time of birth, and other important information on a patient's forward care plan interface. All the data included in the family profile will be linked to an automated message system (enabled through City P hospital's specific generative AI system). The automated message system will be structured to send important reminders, milestones, and confidence boosters to parents as they experience the care journey for their newborns in the NICU and at home.

The digital health modalities used in the CO‐PARTNER parent health engagement system are applied to support processes of personalization and access. Process evaluation should include processes of personalization‐shared decision‐making and tailored care (see Figure 5) and access‐provision of information (see Figure 5). Personalization processes are enabled through using parent‐initiated and flexible streams of communication (remote, video calls, asynchronous SMS messaging, and email) and tailored feedback and on‐demand tailored feedback, resources, and information. Access processes are supported through the digital modalities of on‐demand information and resources, and remote communication functionalities of mobile and Web Apps. It is the recommendation from the parent engagement committee for quality improvement that process evaluation measures should be designed and implemented for shared decision making, tailoring care, and provision of information.

### 5.3 Step 3: Co‐Creation During Real‐Life Testing

**Figure 6:**
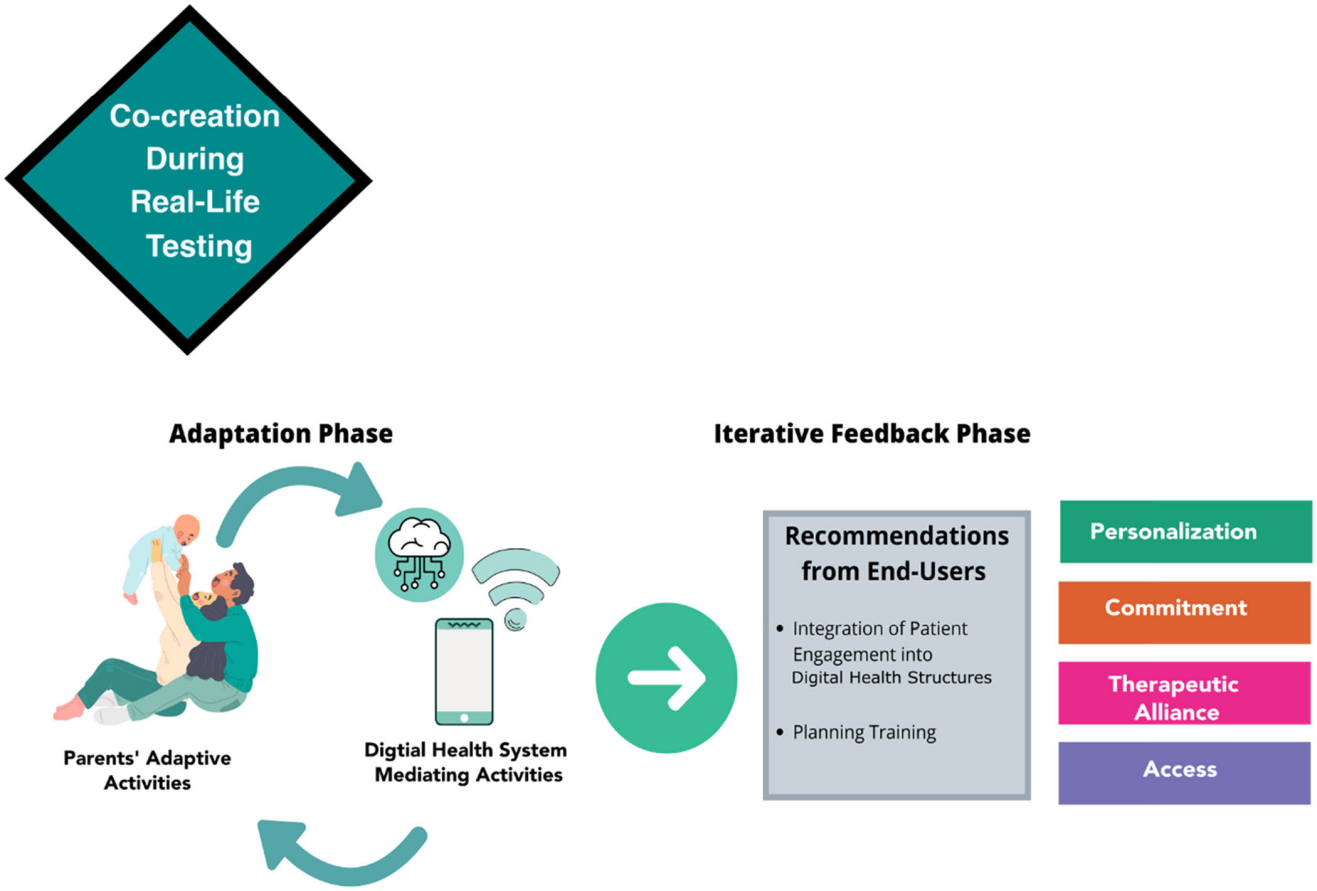
Co‐creation during real‐life testing.

The co‐creation of perinatal digital health systems emerges as parents and technologies interact. New and expectant parents carry out activities during their adaptation/habit formation with real‐time use, and the technology has mediating functions, together a pattern of use will be determined. As noted in the pilot study of a self‐monitoring perinatal digital health system, a socio‐materiality perspective supports the examination of user‐technology interactions (Auxier, Savolainen, et al. 2023). User‐technology interactions inform the types of processes that occur between the parent users and the technology during the co‐creation of new digital health systems. From the purposive literature sample, the elements of co‐creation through real‐life use of perinatal digital health systems were observed and can be illustrated in a narrative description of Step 3. Adaptive and iterative phases under Step 3 were articulated from the interpretation of the scoping review and pilot study of the larger exploratory study and are as follows: (1) The adaptive phase (described through socio‐materiality perspectives) and (2) The iterative feedback phase (described as receiving feedback after use for the refinement and development of digital health system structures and end‐user training) (Auxier, Savolainen, et al. 2023) (see Figure 6).

In the adaptive phase, perinatal digital health users' adaptive activities were related to their personal preferences, attitudes about technology, and level of motivation for improving or maintaining their health during pregnancy or after the birth of their newborns. The mediating activities of technologies manifested throughout digital health systems (*N* = 59; see Table 2) provided new meaningful ways in seeing technology as a partner in perinatal nursing care processes. Co‐designing of perinatal digital health systems was reported as a priority to development in 19 of the included programs (32.2%). Although this was a theme from the available reports, there are many programs that are not using or not reporting patient and public involvement approaches to design. When co‐designing was included, it was important to receive feedback that would be used in subsequent phases of digital health system deployment.

During the iterative phase of Step 3, feedback should be gathered and must be specific to the context and attitudes present for parents and perinatal nurses (Abbass‐Dick et al. 2017; Danbjørg et al. 2015; Franck and O'Brien 2019). Iterative feedback and cyclic design of systems were core components in studies that focused on designing systems with the user perspective in focus. Feedback was gathered and was specific to context and attitudes present within the workplace and with parent stakeholders (Abbass‐Dick et al. 2017; Danbjørg et al. 2015; Franck and O'Brien 2019).

## 6 Discussion

As digital transformations are being experienced in high‐ and middle‐income countries, the responsibility for the maintenance of wellness as well as the construction of perinatal digital health systems can be in the hands of parents and nurses together. Here, a user‐centered nursing engagement model is provided for the development and design of digital systems that capture the principles of person‐centered perinatal care. By integrating patient engagement into system design right from the outset through a model approach that considers the latent mechanisms of action within the structures and processes of person‐centered perinatal care, stakeholders are given the opportunity not only to contribute their ideas about acceptability and feasibility of systems but are supported to set their experiential knowledge into the action through person‐centered intervention mapping. This model has the potential to support initiatives for integrating perinatal service users' engagement in all stages of system design/development and testing in a way that harnesses nursing critical realist frames, supporting efforts toward more equitable perinatal digital health nursing design (Auxier 2023; Ford‐Gilboe et al. 2018). The model supports examination of perinatal digital system design, development, and quality assurance concretely and through the explicit mention of patient engagement structures, processes, and outcomes.

Perinatal digital health systems are being developed with explicit mention of the need to integrate elements of person‐centered care and patient engagement, however, much of the research on perinatal digital health system design until now has not operationalized commitment and therapeutic alliance, and lacks transparency of process evaluations (Auxier, Bender, et al. 2023). Whereas much work has been conducted to understand access and personalization as outcomes of digital health systems, little has been incorporated into the study designs for the observation and evaluation of all engagement attributes as processes and structures of care systems (Auxier, Bender, et al. 2023). Health organizations, researchers, and clinicians are aiming to promote engagement of perinatal health users through access to digital programs, apps, and wearable devices for self‐monitoring procedures, what is needed now, is a sustained focus on developing and monitoring quality with an eye on explicit patient engagement elements (Oberschmidt et al. 2022; Strand et al. 2022). The model described here has the potential to support the co‐construction and sustainment of holistic perinatal digital health systems.

The nursing engagement model is novel. The steps can give researchers and developers a path forward for using person‐centered perinatal digital health development and quality assurance approaches based on theoretical foundations of patient engagement, person‐centered care, nursing quality assurance, and action research. “E‐scaped” care, as described by Nettleton (2004) positions researchers, nurses, and parent users for the creation of new processes within perinatal care practices, without explicit mention of engagement attributes during the design work it is easier to follow the pressures of innovation and advancement of technology and miss nuanced processes important for wholistic and engaging perinatal nursing. The model proposed here can help to harness what Nettleton coined as, the “existing processes of transformation” in relation to the digital transformations (Nettleton 2004). The interactions parents have with digital health modalities could impact how they relate and think about the role of their perinatal health professionals. For example, with new access to information and within their own powerful roles as health users, some parents might resist the connection with their healthcare professionals (Nettleton 2004). By highlighting therapeutic alliance as a core component in perinatal digital health development and quality assurance, it is possible to understand how to better sustain person‐centered care, reciprocity, and partnership within the perinatal nursing practice processes and structures.

Fidelity of perinatal digital health systems is an important area of inquiry; it is confronting for researchers to examine fidelity through the lens of patient engagement because the merit of sustaining fidelity in digital health systems might be questioned in support of more flexible and tailored approaches. Supporting adaptability of digital health systems over fidelity in specific aspects of system structures has been shown to improve health user's perceptions of tailored care (Auxier et al. 2022; O'Brien et al. 2013; Gibson et al. 2021). Disenfranchised groups have unequal access and opportunities for the use of digital services, and some individuals prefer using non‐digital modalities for their healthcare experience (e.g., in‐person visits, analog forms of services). The balance between adaptability and fidelity can be guided through observational studies of practices/adaptations during real‐time testing of systems that focus on engagement principles. Disenfranchised group norms and needs can be integrated into the design and development of perinatal health systems through real‐time observational pilot studies (Oelhafen 2024; Auxier et al. 2022; Auxier, Savolainen, et al. 2023). Further, specific individual differences in preference for use and engagement levels can also be captured.

Step 1 of the model focuses on mapping digital health solutions based on digital modalities that have been shown to support access, personalization, commitment, and therapeutic alliance. Step 2 supports stakeholder and perinatal digital health users' preferences, attitudes, and values through exploration of vignettes and user‐case scenarios (inclusive of descriptions of perinatal digital health nursing structures and processes). During Step 2, process evaluation plans should be developed together with stakeholders based on the patient engagement processes deemed priorities. Step 3 supports a re‐thinking about planning interventions from the perspective of real‐life testing and co‐creation with a focus on patient engagement attributes and human‐technology interaction principles.

### 6.1 Person‐Centered Perinatal Intervention Mapping

A scoping review conducted ahead of this interpretive synthesis revealed that out of 56 different perinatal digital health systems, 53.6% (*n* = 30) had unique combinations of digital health modalities built into the structure (Auxier, Bender, et al. 2023). We were then able to map meaningful use of digital health modalities to patient engagement attributes (Figure 3). Most studies in the interpretive synthesis (*n* = 86) report that digital health in general has important benefits for society, health, and improving engagement of health users but do not explain the unique reasons for using specific digital health modalities from a theoretical perspective. More work should be completed to understand the mediating factors of digital health modalities on the processes related to access, personalization, commitment, and therapeutic alliance. Links were able to be made between function and modality use; however, many authors did not explicitly note why certain modalities were used in the design. By mapping digital health modalities to specific health system challenges, mediating factors can be monitored. Until now, a taxonomy and mapping template was missing.

### 6.2 Process Evaluation Through Monitoring of Patient Engagement Practices

Step 2 depicts an example of a vignette for stakeholder design and gives researchers, clinicians, and patient stakeholders a chance to imagine a digital system designed specifically to combat the health system challenges they have experienced. Step 2 is a novel addition to the perinatal nursing digital health field. The literature on patient engagement in person‐centered perinatal digital health rarely operationalizes person‐centered and engaging care practices as processes, and clear process evaluations are even less common (Oberschmidt et al. 2022; Oelhafen 2024; Auxier, Bender, et al. 2023). Access was operationalized in many of the studies as a mediator rather than as a process of obtaining digital health resources and support, and as a concept for capturing fulfillment of intended use volumes (adherence), and frequency of use (e.g., logins). The presence of personalization was mostly understood through proxy measures of satisfaction, having informational needs met, and perceptions of flexibility of the program. Commitment was captured through behavioral change theories, such as adherence, participation over time, and the typology of participation (evaluation of participation characteristics, i.e., high and low participation). Therapeutic Alliance was understood through a theoretical model (Fontein‐Kuipers, Ausems, et al. 2016) and through observation of the frequency of interactions between healthcare professionals and perinatal users.

Patient engagement practices that make up the processes of person‐centered care should be further developed into process measures. The process measures can be used together between perinatal digital health users and professional care givers and can be approached through the integration of process variables in structures of digital log keeping and Ecological Momentary Assessments (EMA). These modalities provide daily features, behaviors, and health parameter data that could support examination of processes of perinatal care at home or in acute settings. This nursing engagement model provides the opportunity to reframe investigations of perinatal digital health systems, not only from the perspective of effectiveness toward improvement of health outcomes but in evaluating actual person‐centered nursing care practices in real‐time. Researchers and policy makers have struggled to innovate digital health systems due to insufficient reimbursement and legislation that support the use of digital health systems (Chaudhry et al. 2006), this model can support perinatal researchers' and nurses' capacity to describe concrete features of the ever increasing complex digital health systems, allowing for richer descriptions of how patient engagement plays a role in wholistic and engaging digital health nursing practices.

### 6.3 Co‐Creation During Real‐Life Testing

Step 3 illustrates the various digital health processes/interactions that occur related to patient engagement. Co‐creation illustrates that development toward meaningful, sustainable, and manageable use of digital health systems will involve a close look at attitudes, values, and habits of users. Researchers exploring digital health have noted that the immediate impact of digital health systems on health outcomes is virtually unknown (van Gemert‐Pijnen et al. 2011). More should be understood about how digital health users choose and experience their interactions within the systems themselves through sociomaterial and critical realist approaches. This nursing engagement model can support systematic inquiry into elements of co‐creation.

### 6.4 Limitations

This model was developed from evidence relevant to high‐ and middle‐income countries, and many of the research studies were not nurse‐led (Auxier, Bender, et al. 2023). The theoretical foundations of the engagement model should be reviewed after nurse‐led action research is conducted with parents, to ensure the clarity of the model's usefulness in various contexts of nursing research. Further, concept development of parent‐partnered perinatal digital health practices should be conducted to inform the model further.

The evidence to support the construction of Step 3 encompasses mainly digital health users who are interested in and value the use of digital health and technology in their daily lives. Applying knowledge about the process of adaptation to digital health systems will be continually limited if researchers do not also investigate the process of resourcing those perinatal users who are resistant to using digital health systems (i.e., counseling on choice for use of digital health services, and providing education to support digital health literacy) (Oelhafen 2024). Future research should be dedicated to those within the population who prefer to opt out of digital health solutions, as there is a paucity of evidence currently available on this topic within the perinatal digital health nursing field.

Some work has been done to understand how disadvantaged or hard‐to‐reach groups might be motivated to use perinatal digital health systems. Due to the influence even low threshold use can have on user commitment, it is important to support access to disadvantaged groups (Cramer et al. 2018; Doherty et al. 2019). As research reveals those with extreme disturbances to their health might benefit greatly by the simple act of making their issues know and applying small changes that have a great impact on the health outcomes and family wellness (i.e., stopping or limiting smoking or drinking alcohol during pregnancy) (van der Wulp et al. 2014; Whitemore et al. 2019). Doherty and colleagues found in a study using an EMA mood tracking perinatal App that women within ethnic minorities were less likely to install the mobile App, but once they did install the App, there was no difference in level of engagement with the system according to ethnic group (Doherty et al. 2019).

## 7 Conclusion

The product of this interpretive synthesis is a nursing engagement model for the careful re‐framing of person‐centered nursing practices that are inherently directive toward engaging and critical realist approaches to system design and quality assurance. In this study, three steps of a model were constructed for the integration of patient engagement into perinatal digital health nursing: (1) Person‐centered intervention mapping; (2) Integration of process evaluation through stakeholder and user consultation; and (3) Co‐creation during real‐life testing. Future participatory action research should employ this engagement model to support an increased capacity for capturing the digital health system processes and structures that are becoming ubiquitous within the perinatal nursing contexts. This engagement model is intended to reshape the familiar digital nursing practices into unfamiliar and nuanced elements of process and structure for nurses, parents, nurse researchers, health organization leaders, and policy makers. Due to the popularity of digital health use and the increased incentives for measuring patient engagement, more work should be done to develop perinatal digital health systems that are, at their core, person‐centered, and capture the nuances of user engagement. This study recommends that understanding the best fit of digital health modalities will support the integration of patient engagement into these systems, which has the potential to support the development of holistic and engaging nursing practices. Planning and developing more process measures that capture patient engagement attributes will help nurses, nurse researchers, and developers understand their progress toward benchmarks of patient engagement and person‐centered care. Finally, testing digital health solutions in real‐life contexts will allow for more personalization and critical realist interpretations of health users and perinatal nurses' practices as they adapt to digital health transformations.

## Conflicts of Interest

The author declares no conflicts of interest.

## Data Availability

The data that support the findings of this study are openly available in UTU PUB at https://www.utupub.fi/bitstream/handle/10024/175289/Annales%20D%201720%20Auxier.pdf?sequence=1&isAllowed=y.
